# Methodological Choices in Muscle Synergy Analysis Impact Differentiation of Physiological Characteristics Following Stroke

**DOI:** 10.3389/fncom.2017.00078

**Published:** 2017-08-31

**Authors:** Caitlin L. Banks, Mihir M. Pai, Theresa E. McGuirk, Benjamin J. Fregly, Carolynn Patten

**Affiliations:** ^1^Neural Control of Movement Lab, Malcom Randall VA Medical Center Gainesville, FL, United States; ^2^Department of Biomedical Engineering, University of Florida Gainesville, FL, United States; ^3^Rehabilitation Science Doctoral Program, Department of Physical Therapy, University of Florida Gainesville, FL, United States; ^4^Department of Mechanical and Aerospace Engineering, University of Florida Gainesville, FL, United States

**Keywords:** muscle synergies, motor modules, stroke, locomotion, EMG, variability

## Abstract

Muscle synergy analysis (MSA) is a mathematical technique that reduces the dimensionality of electromyographic (EMG) data. Used increasingly in biomechanics research, MSA requires methodological choices at each stage of the analysis. Differences in methodological steps affect the overall outcome, making it difficult to compare results across studies. We applied MSA to EMG data collected from individuals post-stroke identified as either responders (RES) or non-responders (nRES) on the basis of a critical post-treatment increase in walking speed. Importantly, no clinical or functional indicators identified differences between the cohort of RES and nRES at baseline. For this exploratory study, we selected the five highest RES and five lowest nRES available from a larger sample. Our goal was to assess how the methodological choices made before, during, and after MSA affect the ability to differentiate two groups with intrinsic physiologic differences based on MSA results. We investigated 30 variations in MSA methodology to determine which choices allowed differentiation of RES from nRES at baseline. Trial-to-trial variability in time-independent synergy vectors (SVs) and time-varying neural commands (NCs) were measured as a function of: (1) number of synergies computed; (2) EMG normalization method before MSA; (3) whether SVs were held constant across trials or allowed to vary during MSA; and (4) synergy analysis output normalization method after MSA. MSA methodology had a strong effect on our ability to differentiate RES from nRES at baseline. Across all 10 individuals and MSA variations, two synergies were needed to reach an average of 90% variance accounted for (VAF). Based on effect sizes, differences in SV and NC variability between groups were greatest using two synergies with SVs that varied from trial-to-trial. Differences in SV variability were clearest using unit magnitude per trial EMG normalization, while NC variability was less sensitive to EMG normalization method. No outcomes were greatly impacted by output normalization method. MSA variability for some, but not all, methods successfully differentiated intrinsic physiological differences inaccessible to traditional clinical or biomechanical assessments. Our results were sensitive to methodological choices, highlighting the need for disclosure of all aspects of MSA methodology in future studies.

## Introduction

Muscle synergy analysis (MSA) is a mathematical strategy developed under the premise that complex patterns of muscle activity are driven by a small set of activation components termed synergies. Nikolai Bernstein first proposed the idea of synergies to explain how the nervous system simplifies control of a vast number of independent parameters (Bernstein, 1967). While the relationship between the underlying neuromuscular control strategies and the mathematical concept of synergy analysis are still under debate, MSA is arguably an effective method for reducing the dimensionality of a data set into units that represent most of the variability in the original signals. Importantly, there are numerous analytical parameters involved in MSA. *A priori* decisions and assumptions regarding MSA may influence the outcomes and interpretation of results. Among these choices are: number of synergies used, filtering parameters, electromyogram (EMG) normalization method, computational algorithm, output variable normalization method, and which components [i.e., synergy vectors (SVs) or neural commands (NCs)] remain constant and which can vary between trials. Many research groups base these methodological decisions on the intended application of the synergy analysis [e.g., a musculoskeletal model, a device controller, or a comparison to kinematic, kinetic, or functional variables within and across populations (Ivanenko et al., 2003; Bowden et al., 2010; Berger and d'Avella, 2014; Walter et al., 2014)]. Importantly, when comparing studies that utilize MSA, great care is required to understand the consequences of these decisions. Methodological inconsistencies in performing MSA create challenges not only for interpreting results but also replicating analyses across research groups. These inconsistencies limit our ability to build a body of evidence based on this analytical approach and detract from resolving debate regarding the physiological relevance of MSA.

MSA decomposes EMG activation patterns into a smaller dimension of time-varying signals, often referred to as neural commands, and a matrix of weights, or synergy vectors, that can be linearly combined to reconstruct the original EMG signals. Neural commands are sets of basis functions that represent the time-varying component of the signal and are also known as activation components or activation signals (Ivanenko et al., 2005; Cappellini et al., 2006; Gizzi et al., 2011). Synergy vectors are scalar values that represent activity patterns across all EMG signals and are also referred to as m-modes or weighting coefficients (Ivanenko et al., 2005; Ting and Chvatal, 2010). Collectively, one synergy vector and its corresponding neural command can be termed a synergy or module (Clark et al., 2010; Ting and Chvatal, 2010). The number of synergies selected to represent a data set typically stems from the percentage of variance (or variability) accounted for (VAF) by a combination of synergies. Variability is a key component of MSA because the synergies must be flexible enough to combine into the variable movement patterns that humans or animals employ to perform a task. Several numerical methods can be applied to perform MSA decomposition, including principal component analysis (PCA), independent component analysis (ICA), and non-negative matrix factorization (NNMF). PCA is a linear eigenvalue decomposition technique that finds a set of orthogonal components that represent the covariance of the original data set (Chau, 2001). ICA is a non-linear blind-source separation technique that identifies the statistically independent sources that can be re-combined to generate a mixed set of signals (Bell and Sejnowski, 1995; Hart and Giszter, 2004). Non-negative matrix factorization creates a parts-based representation of the final signal using only positive, additive components (Lee and Seung, 1999). Once the choice of analysis method has been made, the corresponding analysis parameters should be carefully chosen based on the intended outcomes.

Myriad methodological choices are required throughout the MSA process. First, EMG signals are processed, typically involving filtering, time normalization, and amplitude normalization. Filtering and amplitude normalization strategies vary greatly in the literature (Ivanenko et al., 2005; Hug et al., 2012; Santuz et al., 2016). Prior to or during the analysis, depending on the algorithm, a decision must be made regarding the number of synergies needed to reconstruct an intended activity. Some investigators specify a minimum percent VAF (or *R*^2^) threshold across all muscles (Roh et al., 2012; Routson et al., 2013), while others include additional criteria such as the requirement that the addition of one more synergy will not increase the VAF by a considerable amount (Ting and Chvatal, 2010; Hayes et al., 2014). In some cases investigators take additional steps, such as measuring the slope of the VAF or *R*^2^ curve and adding a synergy if doing so leads to a substantial change in either of these parameters (Gizzi et al., 2011; Frère and Hug, 2012; Berger and d'Avella, 2014). Additionally, the SVs can be held constant or allowed to vary from trial-to-trial (Frère and Hug, 2012). Finally, after the synergy algorithm is complete, the SVs or NCs are typically normalized in some fashion to facilitate comparisons in the final output.

MSA has a wide range of applications, from musculoskeletal modeling to complementing a biomechanical analysis, and more recently to studying characteristics of movement pathology. Clark et al. showed that fewer synergies could be used to account for muscle activity patterns during walking in the paretic leg of stroke survivors compared to the non-paretic leg or either leg of healthy controls (Clark et al., 2010). Based on these results, they hypothesized that some of the synergies employed by healthy individuals may be merged in the paretic leg of persons following stroke (Clark et al., 2010). This merging of modules was also described in the upper extremity (Cheung et al., 2012) and lower extremity following stroke, incomplete spinal cord injury, and Parkinson's disease, respectively (Rodriguez et al., 2013; Routson et al., 2013; Hayes et al., 2014). However, two other studies reported no difference in the number of synergies between stroke survivors and healthy controls during walking (Gizzi et al., 2011) or in studying the upper extremity (Roh et al., 2012). This variation in results could be attributable to many factors, including: synergy analysis methods, number and choice of muscles included, task performed, chronicity of pathologic condition, and heterogeneity of deficits inherently present following stroke. The latter two details are difficult to control, but careful selection of synergy analysis methods should improve our ability to replicate or compare results across studies. In the absence of repeatable results both within and across clinical populations, it becomes a challenge to understand the significance and utility of MSA.

Recently, several studies have investigated key methodological details involved in MSA, ranging from EMG collection and processing to the chosen factorization algorithm. Selecting the largest and most dominant muscles within a movement synergy decreases the effect of experimental constraints on the outcome of synergy analysis (Steele et al., 2013). The choice of high-pass and low-pass filter cutoff frequencies impacts the number of synergies selected and the quality of reconstruction of the original signals (Hug et al., 2012; Santuz et al., 2016). The number of trials, as well as whether trials are analyzed individually, averaged, or concatenated into a single matrix, has not been found to produce a major impact on the number of synergies extracted, but averaging or concatenating smaller data sets decreases reconstruction quality (Oliveira et al., 2014). Some studies have compared factorization algorithms (Ivanenko et al., 2005; Tresch et al., 2006), while another has focused on comparing variations of the NNMF algorithm (Devarajan and Cheung, 2014). Collectively, researchers are moving toward standardizing synergy analysis methods in an effort to advance the field and allow for better validation of the utility of MSA in context.

Here our goal was to assess how variations in MSA methodology affect the quantification of trial-to-trial variability in muscle synergies. We chose to quantify variability because of Bernstein's theories regarding the importance of variability within the nervous system; while variability is always present in cyclical movements like walking, this movement variability likely arises from the same movement synergy (Bernstein, 1967). We applied MSA to EMG data collected during assessments prior to an experimental rehabilitation intervention that targeted walking recovery in chronic stroke. While the primary outcome, walking speed, improved overall following intervention, the cohort was divided equally between responders (RES) and non-responders (nRES) (Clark and Patten, 2012). Importantly, at baseline no clinical or neuromechanical gait parameter differentiated individuals who were identified as RES and nRES post-intervention. These data, having a known functional outcome, afford an ideal test-bed for analyzing the impact of variations in MSA methodology. Specifically, we sought to determine the ability of MSA to differentiate RES and nRES using only pre-treatment data. We applied MSA to the five greatest and five least treatment responders with useable datasets to evaluate differences in synergy vector and neural command variability. We hypothesized that one or more methods of MSA would detect differences in synergy variability between RES and nRES.

## Methods

This study involves a subset analysis of subjects with chronic hemiparesis following stroke who participated in 8 weeks of rehabilitation. The intervention consisted of 5 weeks of paretic lower extremity power training and 3 weeks of traditional clinic-based gait training (Clark and Patten, 2012). At baseline and post-treatment, self-selected walking speed and EMG data were collected while participants walked over three force plates (Advanced Mechanical Technology, Inc., Watertown, MA). Gait events of heel strike and toe-off were recorded (200 Hz) using a vertical ground reaction force threshold (*F*> 20 N) and target pattern recognition from heel marker placement using a seven-camera Qualisys motion capture system (ProReflex MCU 240, Göteborg, Sweden). Analog force signals were low-pass filtered (second order bidirectional Butterworth, 10 Hz cutoff). Marker data were low-pass filtered (second order bidirectional Butterworth, 6 Hz cutoff). Surface EMG data were sampled (1 kHz) from eight paretic leg muscles: tibialis anterior (TA), medial gastrocnemius (MG), soleus (SO), rectus femoris (RF), vastus lateralis (VL), biceps femoris (BF), semitendinosus (ST), and gluteus medius (GM) using active, pre-amplified electrodes (17 mm inter-electrode distance, input impedance> 100,000,000 Ω, CMRR> 100 dB at 65 Hz, and signal bandwidth 20–3,500 Hz; MA-411, Motion Lab Systems, Baton Rouge, LA). All procedures were approved by the Stanford University Administrative Panels on Human Subjects Research and conducted in accordance with the Declaration of Helsinki.

Participants were classified as either RES or nRES based on post-treatment change in self-selected walking speed. Individuals demonstrating a post-treatment change exceeding a minimal important difference of 0.123 m/s were classified as treatment RES (*n* = 15). Conversely, individuals who did not produce or exceed this change were classified as nRES (*n* = 17). Importantly, clinical and functional measures at baseline were not different between the RES and nRES in the original cohort (Clark and Patten, 2012). The five highest RES and five lowest nRES with useable EMG data sets were selected for the primary analysis. To evaluate the validity of our findings, we compared five additional nRES to the five lowest nRES. The number of trials included for each subject ranged from 2 to 12, with a mean of 10 trials. Subject characteristics for each group can be found in Table 1.

**Table 1:** Subject demographics.

	**Responders (RES)**	**Non-responders (nRES)**	**Validation nRES**
**DEMOGRAPHICS**			
*n*	5	5	5
sex (m/f)	4/1	3/2	4/1
age (yrs)	56.4 ± 6.97	65.5 ± 5.83	66.5 ± 9.79
self-selected walking speed (m/sec)	0.46 ± 0.23	0.33 ± 0.24	0.41 ± 0.32
post-treatment walking speed change (m/sec)	0.26 ± 0.08	0.03 ± 0.03^*^	0.06 ± 0.03^*^
chronicity (mos)	10 ± 3.14	12.8 ± 3.27	15.2 ± 2.34
affected side (r/l)	2/3	0/5	4/1
**CLINICAL CHARACTERISTICS**			
Fugl-Meyer Synergy Subscore (/22)	16 (14,21)	14 (6,18)	16 (3,21)

*Demographic and clinical data are presented mean ± SD and median (range), respectively*.

* *Indicates a significant difference from RES, p < 0.05*.

### Muscle synergy analysis

We used non-negative matrix factorization (NNMF) to perform the MSA for all conditions (Lee and Seung, 1999; Ivanenko et al., 2005; Ting and Chvatal, 2010). All EMG data were band-pass filtered (fourth-order zero phase-lag Butterworth filter, cutoff 20–200 Hz), demeaned, rectified, low-pass filtered (fourth-order zero phase-lag Butterworth) with a variable cutoff frequency of 7/gc Hz (gc corresponds to the duration of the subject's average gait cycle) and time interpolated using the gait events to obtain 101 points per gait cycle (Clark et al., 2010; Chvatal and Ting, 2013; Routson et al., 2013). An individual's ideal low-pass filter frequency relates to the frequency of the task performed, which is variable in this sample because subjects walked overground at self-selected speed (Shiavi et al., 1998; Hug, 2011; Meyer et al., 2016).

We performed MSA using a total of 30 methodological variations comprised of: five approaches for EMG normalization, two approaches for SV calculation, and three approaches for synergy output normalization. The five EMG normalization approaches were: unit magnitude per trial (MagPer), maximum value over all trials (MaxOver; Clark et al., 2010; Frère and Hug, 2012; Routson et al., 2013; Zelik et al., 2014), maximum value per trial (MaxPer; Gizzi et al., 2011; Walter et al., 2014), unit variance per trial (UnitPer), and unit variance over all trials (UnitOver; Roh et al., 2012; Steele et al., 2013). The SV calculation approaches either held SVs constant across all trials (Clark et al., 2010; Ting and Chvatal, 2010) or allowed them to vary (Ivanenko et al., 2005; Cappellini et al., 2006). The three synergy output normalization approaches were: SVs by unit magnitude (SV Mag)^1^, SVs by maximum value (SV Max; Safavynia and Ting, 2012; Chvatal and Ting, 2013; Rodriguez et al., 2013), and NCs by maximum value (NC Max; Ivanenko et al., 2005; Gonzalez-Vargas et al., 2015). If SVs were normalized, then NCs were multiplied by the same normalization values so that their product remained constant, and vice versa. Every possible combination of EMG normalization, SV calculation, and synergy output normalization was applied, thus creating 30 different methodological variations of MSA.

EMG normalization was either computed within individual trials (per trial) or across all trials within a given muscle (over all trials). MagPer normalization involves dividing each element within the vector of 101 EMG data points by its 2-norm, to create a unit vector:

(1) $$y_{MagPer}=\frac{1}{\left||x|\right|}x,$$

where ***x*** is the original EMG vector and ***y*** is the normalized EMG vector.

In MaxPer normalization, each vector element is divided by the vector's maximum value:

(2) $$y_{MaxPer}=\frac{1}{max(x)}x$$

MaxOver normalization involves the same calculation as MaxPer except the denominator is replaced with the maximum EMG value for the given muscle over all walking trials. UnitPer normalization involves dividing each element of the EMG vector by the vector's standard deviation:

(3) $$y_{UnitPer}=\frac{1}{std(x)}x$$

Similarly to MaxOver, UnitOver involves dividing the EMG vector elements by the standard deviation over all trials for a given muscle within a subject.

After each iteration of NNMF, the calculated synergies were sorted within each trial, since NNMF algorithms do not output synergies in any particular order. The neural commands were sorted using the maximal cosine similarity (cos_*sim*_):

(4) $${\text{cos}}_{sim}\left(a,b\right)=\cos\theta_{ab}=\frac{a\cdot b}{\left||a||||b|\right|},$$

where ***a*** and ***b*** are two neural commands within a synergy and θ is the angle between the two vectors. This step was performed to ensure that each synergy was similar across trials within each subject (d'Avella and Bizzi, 2005; Santuz et al., 2016).

Once MSA was completed for each subject, we calculated the trial-to-trial variability in the SVs and the trial-to-trial similarity in NCs as a basis for identifying differences between RES and nRES at baseline. VAF was averaged within subjects to quantify mean differences between RES and nRES. Standard errors in the SVs were averaged within and across subjects for each of the RES and nRES to quantify the variability within these outcome measures. Neural command similarity was calculated two ways: using the cosine similarity and the maximum value of the circular cross-correlation coefficient. The cosine similarity was calculated as in Equation (4), above, for every combination of trials within each synergy (d'Avella and Bizzi, 2005; Coscia et al., 2014; Santuz et al., 2016). These values were then averaged to determine the trial-to-trial similarity of each NC. The cross-correlation coefficient was also averaged across all possible combinations of trials (Ivanenko et al., 2004; Frère and Hug, 2012).

A two-way group^*^synergy analysis of variance (ANOVA) was conducted on mean VAF across all methods. Tukey's HSD was applied *post-hoc* to assess significant effects. Hedges' *g-*test was used to compare trial-to-trial differences in the SVs, NCs, and VAF in the RES and nRES across MSA methods. Hedge's g is complementary to the *t*-test, but due to the small sample size and the desire to determine the generalizability of results to larger data sets, we computed effect sizes rather than performing inferential statistics. We interpret results using effect thresholds as follows: small = ±0.2, medium = ±0.5, large = ±0.8, very large = ±1.3 (Cohen, 1977). Because the present study is a test-bed for MSA, we considered medium effect sizes and larger noteworthy, with special emphasis on large and very large effects. To evaluate our results, we also calculated effect sizes of noteworthy MSA methods between the five worst nRES and five additional nRES from the larger dataset.

EMG processing, MSA, and Hedge's *g-*tests were performed using Matlab's Optimization Toolbox™ (Release r2015a, The MathWorks, Natick, MA) and the Measures of Effect Size Toolbox (Hentschke and Stüttgen, 2013). Custom functions were written to analyze these data using each of the MSA methods described above. ANOVA and *post-hoc* analyses were conducted in JMP Pro 11 (SAS Institute, Inc., Cary, NC, USA).

## Results

**Figure 1:**
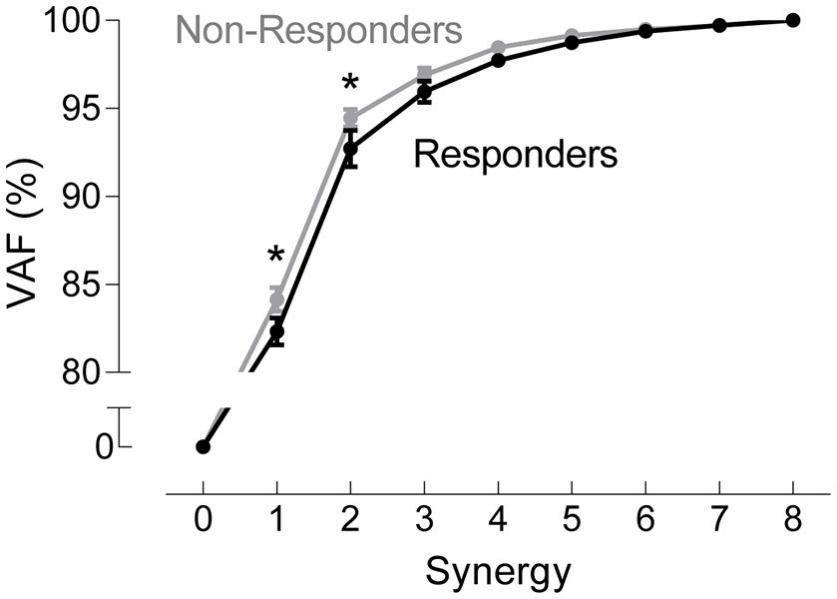
Pre-treatment variance accounted for (VAF). VAF for each synergy, averaged across all muscle synergy analysis methods for non-responders (gray) and responders (black). Data represent mean ± standard error across subjects.

**Figure 2:**
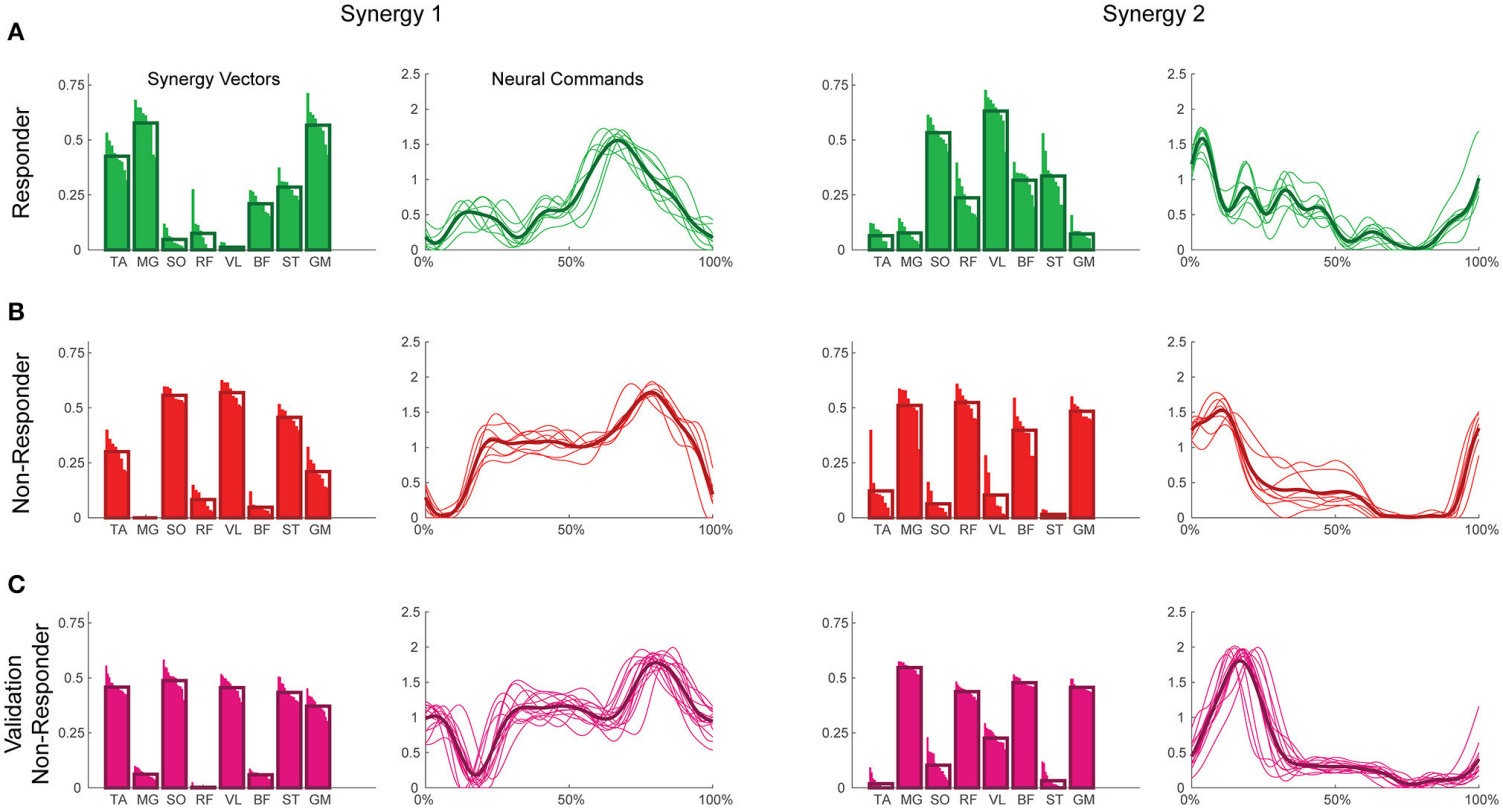
Example synergies for a responder **(A)**, non-responder **(B)**, and validation non-responder **(C)**. The left two columns depict Synergy 1, while the right two depict Synergy 2. Within each synergy, the leftmost plot represents the synergy vectors (SVs) and the rightmost plot represents the neural commands (NCs). Individual trials are represented by thin bars (SVs) and thin lines (NCs), while the thicker bars and lines represent the average across trials for each subject. Synergies were extracted using maximum value per trial EMG normalization, varying SVs, and normalization of SVs to unit magnitude. TA, tibialis anterior; MG, medial gastrocnemius; SO, soleus; RF, rectus femoris; VL, vastus lateralis; BF, biceps femoris; ST, semitendinosus; GM, gluteus medius.

**Figure 3:**
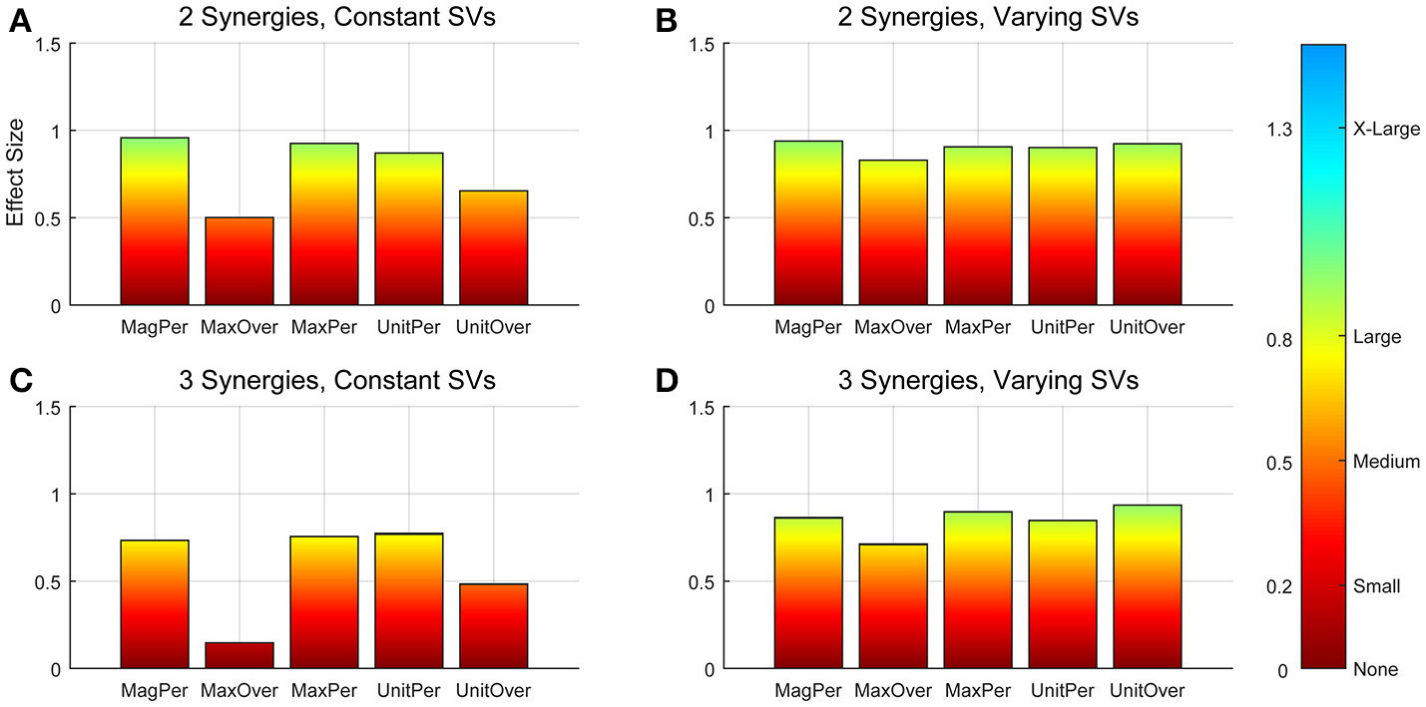
Absolute effect sizes at baseline, measured by variance accounted for (VAF). Effect sizes for two synergies with constant synergy vectors (SVs, **A**), two synergies with varying SVs **(B)**, three synergies with constant SVs **(C)**, and three synergies with varying SVs **(D)**. EMG normalization methods include: magnitude per trial (MagPer), maximum value over all trials (MaxOver), maximum value per trial (MaxPer), unit variance per trial (UnitPer), and unit variance over all trials (UnitOver). Color bar represents effect size values.

A two-way ANOVA for mean VAF revealed significant main effects of group (*p* = 0.005) and synergy (*p* < 0.0001) but no interaction effect. RES had a lower average pre-treatment VAF than did nRES (Figure 1). Across all methods, only two synergies were needed to reach at least 90% VAF (92.72 ± 1.04% for RES, 94.44 ± 0.50% for nRES, mean ± standard error). Figure 2 shows example synergies for one RES and one nRES (top two rows). Addition of a third synergy increased the average VAF to 95.95 ± 0.61% for RES and 96.90 ± 0.40% for nRES. Whether an absolute 90% VAF cutoff or an average 95% requirement was applied, the full EMG data set could be well-approximated using either two or three synergies. Since VAF is unaffected by output normalization, there are 10 possible combinations of MSA methods for comparing VAF between groups. With two synergies, all 10 methods produced at least medium effects, indicating that a larger sample with similar characteristics is likely to have significantly greater VAF for the non-responders than the responders (Figure 3). For three synergies, there was one small effect, four medium effects, and four large effects. All of the large effects occurred with varying SVs. Because of these differential effects in VAF, we examined both two and three synergies for effects in SV and NC variability.

**Figure 4:**
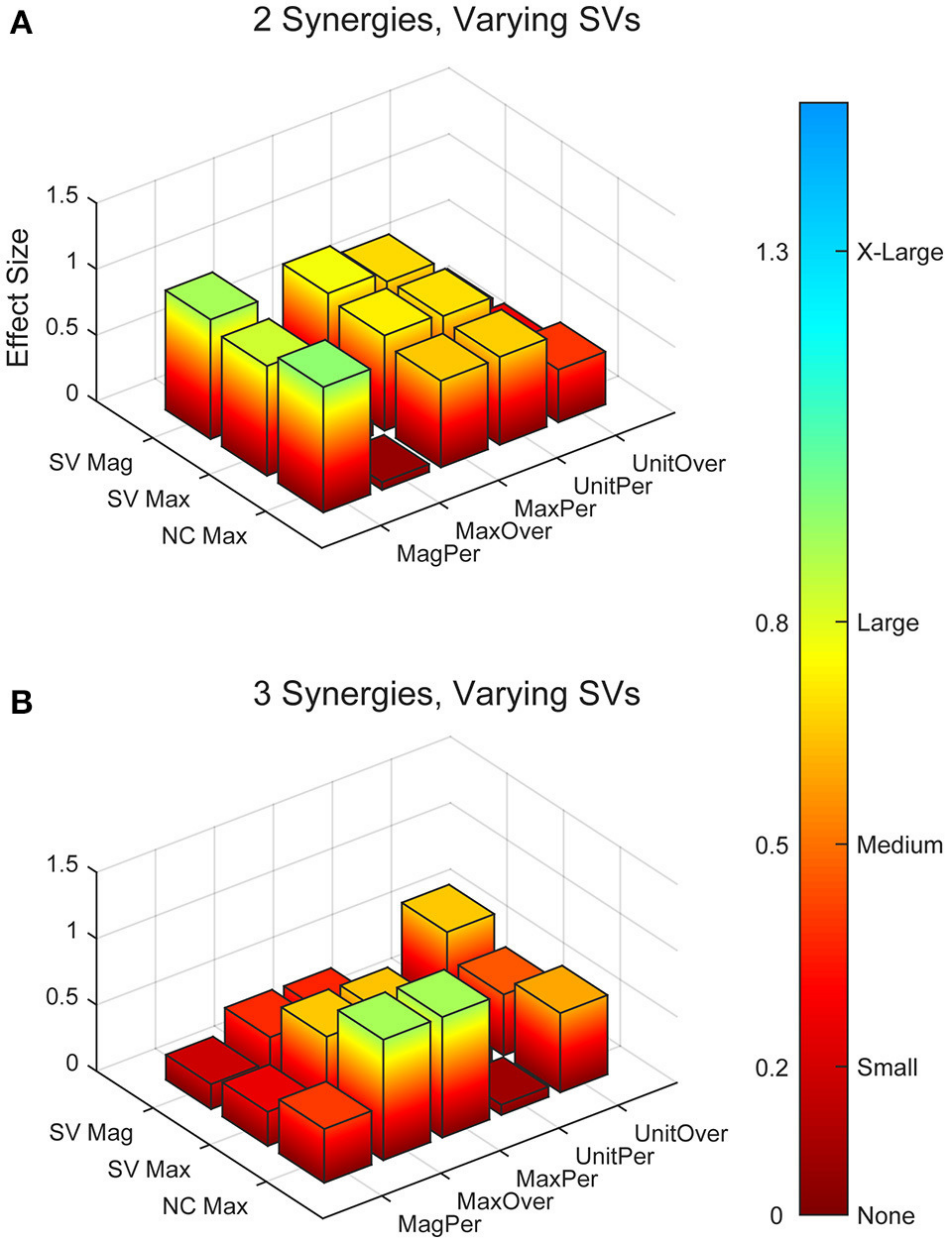
Absolute effect sizes at baseline for synergy vector (SV) variability. Effect sizes for two varying synergies **(A)** and three varying synergies **(B)**. EMG normalization methods include: magnitude per trial (MagPer), maximum value over all trials (MaxOver), maximum value per trial (MaxPer), unit variance per trial (UnitPer), and unit variance over all trials (UnitOver). Output normalization methods include SVs by magnitude (SV Mag), SVs by maximum value (SV Max), and neural commands by maximum value (NC Max). Color bar represents effect size values.

Synergy analysis methodology greatly influenced the calculated trial-to-trial variability within the synergy vectors. With two synergies and varying SVs between trials, six methods produced medium effects and three methods produced large effects (Table 2). All of these effects were positive, indicating greater variability in the RES than the nRES. Figure 4 illustrates absolute effect sizes when comparing SV variability between RES and nRES with two and three varying synergies. MagPer EMG normalization revealed large effects with two synergies regardless of SV output normalization method. For three synergies, notable differences between RES and nRES were revealed with six methods. The greatest effect sizes were revealed with MaxPer and MaxOver EMG normalization and NCs normalized to their maximum value.

**Table 2:** Notable effects in synergy vector variability.

**Synergies**	**EMG normalization method**	**Output normalization method**	**Effect size^a^**
2, Varying SVs	MagPer	SV Mag	0.904
		SV Max	0.830
		NC Max	0.949
	MaxPer	SV Mag	0.762
		SV Max	0.720
		NC Max	0.655
	UnitPer	SV Mag	0.680
		SV Max	0.697
		NC Max	0.665
3, Varying SVs	MaxOver	SV Max	−0.653
		NC Max	−0.909
	MaxPer	SV Max	−0.647
		NC Max	−0.909
	UnitOver	SV Mag	−0.653
		NC Max	−0.597

*SVs, synergy vectors; MagPer, magnitude per trial; SV Mag, synergy vector magnitude; SV Max, synergy vector maximum value; NC Max, neural command maximum value; MaxPer, maximum value per trial; UnitPer, unit variance per trial; MaxOver, maximum value over all trials; UnitOver, unit variance over all trials*.

a *Effect sizes calculated using Hedge's g. Positive values in SV variability indicate greater variability in RES*.

**Figure 5:**
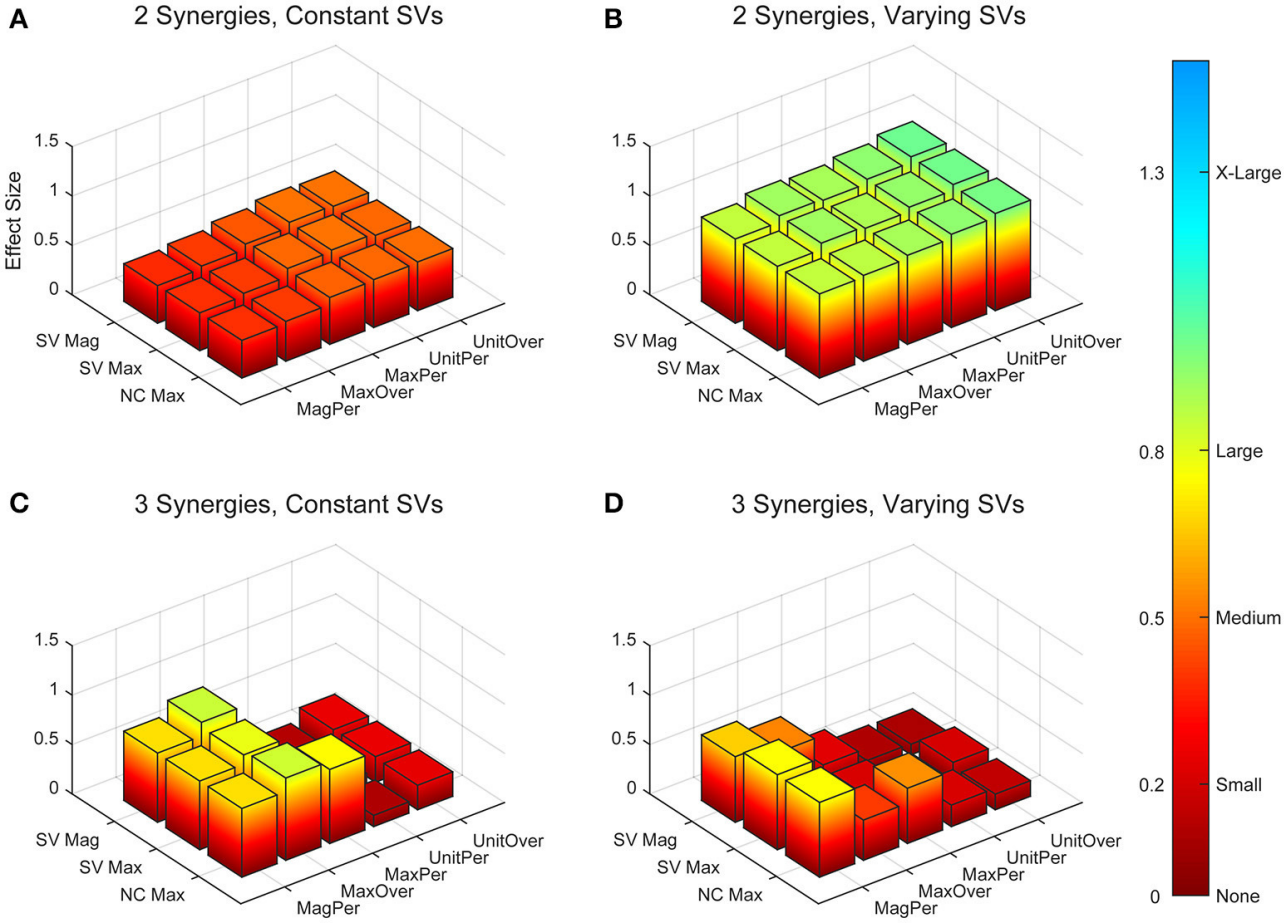
Absolute effect sizes at baseline for neural command (NC) variability as measured by the cosine similarity. Effect sizes are represented for two synergies with constant synergy vectors (SVs, **A**), two synergies with varying SVs **(B)**, three synergies with constant SVs **(C)**, and three synergies with varying SVs **(D)**. EMG normalization methods include: magnitude per trial (MagPer), maximum value over all trials (MaxOver), maximum value per trial (MaxPer), unit variance per trial (UnitPer), and unit variance over all trials (UnitOver). Output normalization methods include SVs by magnitude (SV Mag), SVs by maximum value (SV Max), and NCs by maximum value (NC Max). Color bar represents effect size values.

**Figure 6:**
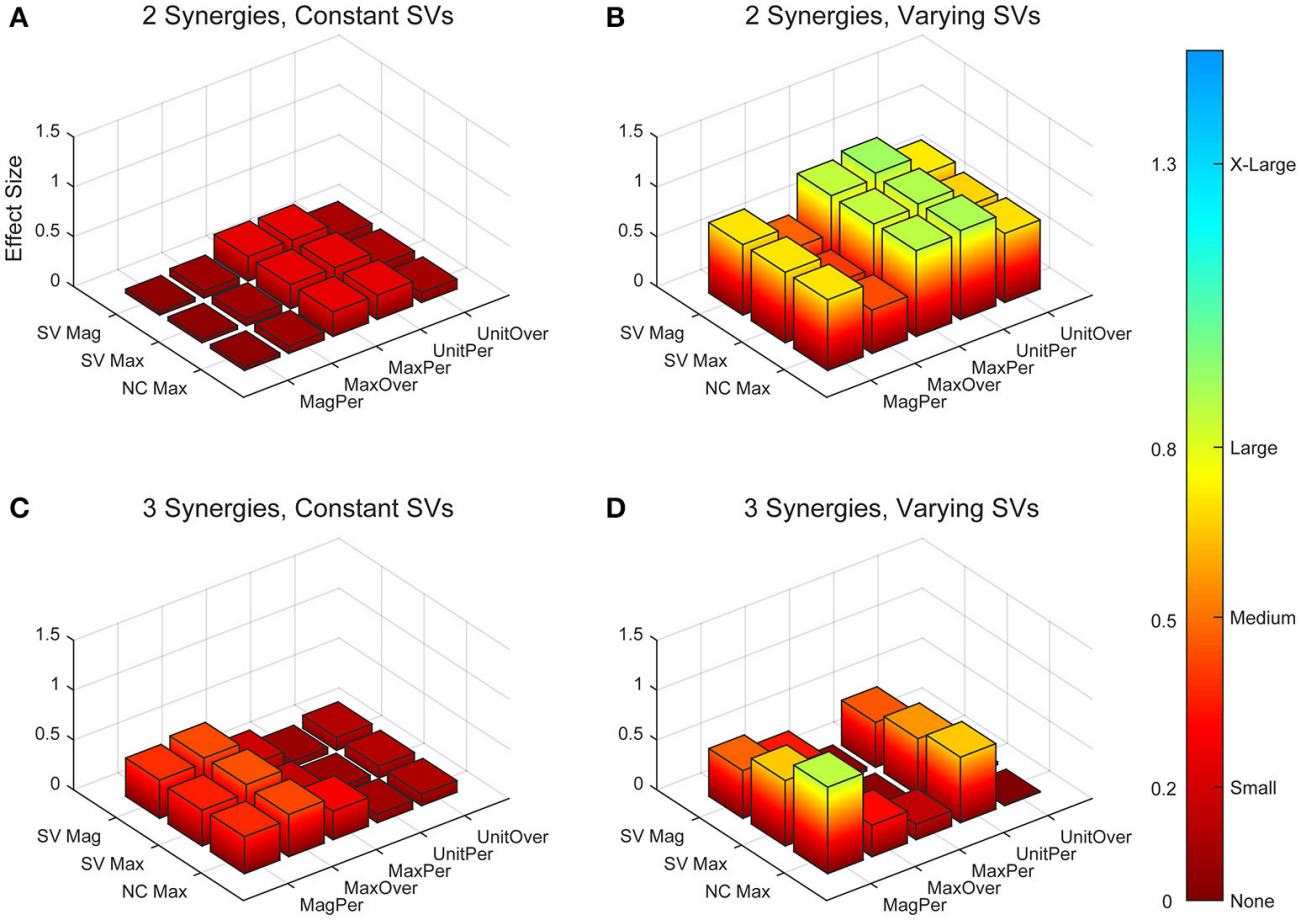
Absolute effect sizes at baseline for neural command (NC) variability measured by cross-correlation. Effect sizes are represented for two synergies with constant synergy vectors (SVs, **A**), two synergies with varying SVs **(B)**, three synergies with constant SVs **(C)**, and three synergies with varying SVs **(D)**. EMG normalization methods include: magnitude per trial (MagPer), maximum value over all trials (MaxOver), maximum value per trial (MaxPer), unit variance per trial (UnitPer), and unit variance over all trials (UnitOver). Output normalization methods include SVs by magnitude (SV Mag), SVs by maximum value (SV Max), and NCs by maximum value (NC Max). Color bar represents effect size values.

The trial-to-trial variability within the neural commands differed depending on both the MSA method and the method of quantifying variability (Table 3). For both cosine similarity and cross-correlation, negative effect sizes indicate greater trial-to-trial variability among RES, compared to nRES. For the cosine similarity metric with two synergies and constant SVs, there were three medium effects and no large or very large effects (Figure 5). Two synergies with varying SVs produced large effects for all fifteen combinations of EMG and output normalization. Three synergies with constant SVs revealed five medium effects and two large effects. Three synergies with varying SVs revealed five medium and no large or very large effects. For the cross-correlation comparison with constant SVs, neither two nor three synergies produced notable effects (Figure 6). Two synergies with varying SVs revealed six medium effects and six large effects (Table 3). Three synergies with varying SVs revealed three medium effects and one large effect. In general, the same patterns were present across output normalization methods but two synergies with varying SVs produced large effects most frequently.

**Table 3:** Notable effects in neural command variability.

**Synergies**	**EMG normalization method**	**Output normalization method**	**Cosine similarity effect size^a^^,^^b^**	**Cross-correlation effect size^a^^,^^c^**
2, Constant SVs	UnitPer	SV Mag	−0.509	–
		SV Max	−0.506	–
	UnitOver	SV Mag	−0.500	–
2, Varying SVs	MagPer	SV Mag	−0.852	−0.707
		SV Max	−0.853	−0.707
		NC Max	−0.851	−0.709
	MaxOver	SV Mag	−0.917	–
		SV Max	−0.917	–
		NC Max	−0.868	–
	MaxPer	SV Mag	−0.902	−0.858
		SV Max	−0.896	−0.851
		NC Max	−0.901	−0.861
	UnitPer	SV Mag	−0.945	−0.912
		SV Max	−0.940	−0.882
		NC Max	−0.943	−0.893
	UnitOver	SV Mag	−1.00	−0.718
		SV Max	−0.998	−0.671
		NC Max	−0.983	−0.693
3, Constant SVs	MagPer	SV Mag	−0.700	–
		SV Max	−0.695	–
		NC Max	−0.695	–
	MaxOver	SV Mag	−0.841	–
		SV Max	−0.789	–
		NC Max	−0.832	–
	MaxPer	NC Max	−0.749	–
3, Varying SVs	MagPer	SV Mag	−0.663	–
		SV Max	−0.759	−0.656
		NC Max	−0.756	−0.868
	MaxOver	SV Mag	−0.539	–
	MaxPer	NC Max	0.558	–
	UnitPer	SV Max	–	−0.566
		NC Max	–	−0.658

*SVs, synergy vectors; MagPer, magnitude per trial; SV Mag, synergy vector magnitude; SV Max, synergy vector maximum; NC Max, neural command maximum; MaxOver, maximum value over all trials; MaxPer, maximum value per trial; UnitPer, unit variance per trial; UnitOver, unit variance over all trials; –, no notable effect*.

a *Effect sizes calculated using Hedge's g. Negative values in NC cosine similarity and NC cross-correlation indicate greater variability in RES*.

b *Neural command similarity compared by trial-to-trial cosine similarity*.

c *Neural command similarity compared by trial-to-trial cross-correlation*.

To validate our findings, we compared results from the original nRES to those from five additional nRES identified from the larger data set. Example synergies for a validation nRES are shown in Figure 2 (bottom row), which appear qualitatively similar to the example nRES in the same figure. Two synergies were sufficient to achieve 90% VAF in all validation nRES across all methods, and there were no differences in VAF between the original nRES and the validation group. Because two synergies with varying SVs produced the most consistent effects in the initial RES/nRES analysis, validation focused on large effects within this subset of results. In this case, large effects in the initial RES/nRES analysis coupled with small or no effects in the nRES validation analysis are desirable findings, indicating that the method has the capacity to differentiate between response groups. For synergy vector variability, MagPer EMG normalization produced large effects in the initial analysis and no effects when comparing nRES to nRES (Table 4). For cosine similarity within the neural commands, MagPer EMG normalization produced medium effects when comparing between nRES and validation nRES across all output normalization methods. All other EMG methods that produced large effects from the initial analysis produced small or no effects in the validation analysis. For NC cross-correlation, all comparisons within the validation analysis revealed small or no effects.

**Table 4:** Comparison of initial results for two synergies with varying synergy vectors with non-responder validation analysis.

**Metric**	**EMG normalization method**	**Output normalization method**	**RES vs. nRES effect size^a^**	**nRES vs. validation nRES effect size^a^**
SV Variability	MagPer	SV Mag	0.904	−0.178
		SV Max	0.830	−0.110
		NC Max	0.949	−0.150
NC Cosine Similarity	MagPer	SV Mag	−0.852	−0.687
		SV Max	−0.853	−0.705
		NC Max	−0.851	−0.695
	MaxOver	SV Mag	−0.917	−0.070
		SV Max	−0.917	−0.101
		NC Max	−0.868	−0.157
	MaxPer	SV Mag	−0.902	−0.361
		SV Max	−0.896	−0.357
		NC Max	−0.901	−0.392
	UnitPer	SV Mag	−0.945	−0.127
		SV Max	−0.940	−0.109
		NC Max	−0.943	−0.106
	UnitOver	SV Mag	−1.00	0.115
		SV Max	−0.998	0.127
		NC Max	−0.983	0.138
NC Cross-Correlation	MaxPer	SV Mag	−0.858	0.040
		SV Max	−0.851	0.038
		NC Max	−0.861	0.050
	UnitPer	SV Mag	−0.912	0.261
		SV Max	−0.882	0.254
		NC Max	−0.893	0.242

*RES, responders; nRES, non-responders; SV, synergy vector; NC, neural command; MagPer, magnitude per trial; SV Mag, synergy vector magnitude; SV Max, synergy vector maximum; NC Max, neural command maximum; MaxOver, maximum value over all trials; MaxPer, maximum value per trial; UnitPer, unit variance per trial; UnitOver, unit variance over all trials*.

a *Effect sizes calculated using Hedge's g. Positive values in SV variability and negative values in NC cosine similarity and NC cross-correlation indicate greater variability in RES*.

## Discussion

This study demonstrates that the methodological choices made during MSA have a significant impact on the outcome. Several, but not all, MSA methods were able to differentiate between two groups of pathologic individuals with no distinguishing clinical differences, but presumed intrinsic physiologic differences. Two synergies with varying SVs across trials produced the highest frequency and greatest magnitude of effects. When assessing SV variability, MagPer EMG normalization was the most salient method within this analysis. However, when comparing NCs by cosine similarity or cross-correlation, any of the EMG normalization methods other than MagPer produced differences between RES and nRES that were validated in a secondary analysis. MSA output normalization had no notable influence on results. In general, our results were highly sensitive to changes in MSA methodology, illustrating the need for careful methodological consideration and disclosure when conducting MSA or comparing with results found in the literature.

### Methodological considerations

We do not believe that our choice of one signal decomposition method (i.e., NNMF) limits the ability to detect group differences within our results. We chose to use NNMF for this MSA for three reasons: muscle activation signals are inherently non-negative, other methods require assumptions such as orthogonality and independence of parameters (Ting and Chvatal, 2010), and NNMF is a commonly used technique, allowing for greater generalizability of results. Previous work using MSA illustrates that NNMF produces synergy vectors and neural commands that are highly correlated with those produced by other computational methods such as PCA and ICA (Ivanenko et al., 2005; Cappellini et al., 2006). NNMF is particularly robust to differences in data distribution and noise across data sets (Tresch et al., 2006). NNMF tends to define a solution subspace where the synergies can be found, building non-negative components (i.e., SVs and NCs) together to reconstruct the original signals (Ting and Chvatal, 2010). This approach is appropriate for the physiological interpretation of the muscle synergy data. There are several variations of the NNMF algorithm and it is possible that changing the algorithm could change MSA results. However, the current consensus in the literature is that the decomposition method does not drastically change the structure of the extracted synergies (Ivanenko et al., 2005, 2006; Cappellini et al., 2006; Tresch et al., 2006). Ultimately, the choice of decomposition method should be tailored to the intended application of the results.

It is common for investigators to specify a minimum VAF cutoff of 90% for identification of the number of synergies that can be used to reconstruct the original signals (Clark et al., 2010; Frère and Hug, 2012; Roh et al., 2012). If this common cutoff was applied to the current analysis, two synergies would have been more than adequate to reconstruct the original EMG walking signals with reasonable accuracy. If a cutoff of 95% VAF for all methods and all subjects was applied, all but one subject would have required three synergies, with the remaining subject requiring four. The decision to use either two or three synergies simplifies the analysis and prevents potential over-fitting problems. This result is also consistent with findings that individuals post-stroke require fewer synergies to reconstruct their movements (Clark et al., 2010; Cheung et al., 2012). However, three varying synergies were also able to detect differences between groups for some methods.

Decisions beyond decomposition method and number of synergies, such as fixing or varying SVs and EMG normalization method, exhibited a profound influence on the ability to detect group differences within our sample. Allowing SVs to vary from trial-to-trial, rather than fixing them across trials, produced more frequent and larger effect sizes, although this choice is less common in the literature (Ivanenko et al., 2005; Oliveira et al., 2014). MagPer EMG normalization produced large differences between groups for SV variability. When comparing NCs with cosine similarity, all methods of output normalization and EMG normalization (except MagPer) produced group differences that were validated with further analyses. When applying a cross-correlation analysis, MaxPer and UnitPer EMG normalization were the best performing methods across all output normalization approaches. EMG normalization is a common step in MSA, however one utilized without much justification for the choice of method. The impact of normalization strategies is shown when one investigator attempts to replicate the analysis of another. Two research groups performed the same experiment with one added condition, but found different results, which could be attributable in part to differences in MSA methods (de Rugy et al., 2013; Berger and d'Avella, 2014). Our analysis indicates that choice of normalization methods and the choice of constant or variable SVs across trials have a strong effect on the outcomes, thus representing a non-trivial choice among MSA parameters.

It is conceivable that variation in the selection of muscles included in the analysis could impact results (Steele et al., 2013). Addition of more muscles or muscles that could more accurately capture the differences between RES and nRES would likely increase the resolution to detect group differences. These data, and other synergy analysis data, should be interpreted within the context of the muscles included in the analysis.

### Detecting physiological differences

Differences in synergy vector and neural command variability between RES and nRES were greatest using two synergies with varying SVs. The largest difference between the two groups was found in the SVs when EMG was normalized to its maximum value per trial, while EMG normalization was less important when detecting differences in the neural commands. The effect sizes indicate that RES had more variability in their SVs than did nRES. Previous findings indicate that the SVs, but not the NCs, reveal differences between individuals post-stroke and healthy controls (Gizzi et al., 2011). Our results differ slightly, indicating that variability in the SVs and NCs can be used to quantify differences between groups after stroke. However, we compared two groups, both with neural pathology. Our most salient results could not have been produced if the SVs were held constant, an assumption that many investigators have used in their analyses (Clark et al., 2010; Ting and Chvatal, 2010). Differences in trial-to-trial neural command similarity between RES and nRES were also greatest when two varying synergy vectors were used. Similar to our findings in the SVs, these effects indicate that RES exhibited more trial-to-trial variability in the NCs than nRES. This characteristic could be due to a greater flexibility of commands that can be combined to produce a richer variety of movement patterns (Bernstein, 1967), which could be relevant to identifying one's capacity for recovery or treatment response. When NCs were compared using cross-correlation instead of cosine similarity, the results were similar; however, the effects were often blunted. This difference in effect magnitude provides further indication regarding the importance of all aspects of MSA with respect to the final results.

### Recommendations for reporting results

MSA has been popularized due to its ability to reduce the dimensionality of complex patterns of muscle activity (Ivanenko et al., 2006). However, the rapid popularization of a technique, coupled with the lack of validation of each MSA methodological choice, presents implementation challenges. In publications utilizing synergy analysis, some investigators provide more than adequate disclosure of the methods employed; however, others provide little to no detail, making comparison of results a challenge. We therefore propose a list of decisions that should be reported explicitly in manuscripts performing MSA:

1. Muscles included in analysis,
2. EMG filtering methods,
3. EMG normalization method,
4. computational method (e.g., NNMF, PCA, ICA, FA),
5. whether constant or varying synergy vectors were used,
6. sorting method (if applicable),
7. output vector normalization method, and
8. synergy comparison method (e.g., cross-correlation)

The present comparison of MSA methods is not intended to suggest there is any one “best” method for all future applications of MSA. Rather, our results illustrate and emphasize the vast differences produced by variations in methodological choices. Ultimately, the choice of analysis methodology should be tailored to the application and research design. To facilitate understanding and reproducibility we recommend disclosure and justification of methods. Such openness between investigators will move research forward, improving the likelihood and timeliness of a research impact involving MSA.

## Conclusions

The main goal of MSA, when applied to pathologic populations, is to better understand the intrinsic physiologic characteristics reflected in muscle activity. MSA, with specific focus on trial-to-trial variability, has the potential to provide insight regarding neural strategies that could be relevant to human performance and rehabilitation. Notably, our analysis revealed large differences between response groups with only 10 subjects. Clinical assessments typically require large samples, lack sensitivity, and in this case, fail to differentiate subpopulations that would later respond to an intervention. If information from MSA can be successfully employed as a predictor of recovery or intervention response on an individual basis, this information could improve outcomes of neurorehabilitation. The analysis must, however, be performed properly, with careful selection and justification of methods.

## Author contributions

BF, CP, and MP conceived and designed experiments; MP, TM, and BF contributed analysis tools; CB and MP performed experiments; CB analyzed data, prepared figures, and drafted manuscript; CB, BF, and CP interpreted results of experiments; CB, BF, and CP revised manuscript; CB, MP, TM, BF, and CP approved final version of manuscript and agreed to be accountable for all aspects of the work.

### Conflict of interest statement

The authors declare that the research was conducted in the absence of any commercial or financial relationships that could be construed as a potential conflict of interest.

## Footnotes

^1^MATLAB. Statistics and Machine Learning Toolbox. Natick, MA: The MathWorks, Inc.

## Abbreviations

MSA: Muscle synergy analysis
RES: treatment responders
nRES: treatment non-responders
SV: synergy vector
NC: neural command
VAF: variance accounted for
NNMF: non-negative matrix factorization
MagPer: magnitude per trial
MaxOver: maximum value over all trials
MaxPer: maximum value per trial
UnitPer: unit variance per trial
UnitOver: unit variance over all trials.
